# Phase I trial of concurrent chemoradiotherapy with docetaxel, cisplatin and 5-fluorouracil (TPF) in patients with locally advanced squamous cell carcinoma of the head and neck (SCCHN)

**DOI:** 10.1038/sj.bjc.6601471

**Published:** 2004-01-20

**Authors:** H Katori, M Tsukuda, I Mochimatu, J Ishitoya, S Kawai, Y Mikami, H Matsuda, Y Tanigaki, C Horiuchi, Y Ikeda, T Taguchi, M Ono, T Yoshida, S Hirose, Y Sakuma, K Yamamoto

**Affiliations:** 1 Department of Otolaryngology, Yokohama City University School of Medicine Medical Center, 3-46 Urafune-chou, Minami-ku, Yokohama 232-0024, Japan; 2 Department of Otolaryngology, Yokohama City University School of Medicine, 3-9 Fukuura, Kanazawa-ku, Yokohama 236-0004, Japan

**Keywords:** chemoradiotherapy, docetaxel, cisplatin, 5-fluorouracil, squamous cell carcinoma of the head and neck (SCCHN)

## Abstract

The aim of this study was to evaluate the efficacy and toxicity of a concurrent chemoradiotherapy using docetaxel, cisplatin and 5-fluorouracil (5-FU) (TPF) in patients with locally advanced squamous cell carcinoma of the head and neck (SCCHN). In total, 19 patients with previously untreated stage III–IV SCCHN were entered onto this trial. Patients received two cycles of chemotherapy. Cycles were repeated every 4 weeks. The starting doses (dose level 1) were docetaxel 60 mg m^−2^, cisplatin 70 mg m^−2^, and 5-day continuous infusion of 5-FU 600 mg m^−2^ day^−1^. Radiation was targeted to begin on the first day of chemotherapy, day 1. The total radiation dose to the primary tumour site and neck lymph nodes was between 63.0 and 74.0 Gy. At least three patients were examined at each dose level before advancing to the next level. The maximum-tolerated dose (MTD) of this regimen was docetaxel 60 mg m^−2^, cisplatin 60 mg m^−2^ and 5-FU 600 mg m^−2^ day^−1^. The main toxicities were mucositis (grade 3 and 4, 79%), leukocytopenia (grade 3 and 4, 53%), neutropenia (grade 3 and 4, 42%), anaemia (grade 3, 16%), liver dysfunction (grade 3, 11%) and renal dysfunction (grade 2, 11%). The overall response rate was 100%, including 84% complete responses (CRs). This concurrent chemoradiotherapy with TPF was safe and well tolerated. The high CR rate justifies further evaluation of this chemoradiotherapy modality in advanced SCCHN patients.

In the majority of patients with squamous cell carcinoma of the head and neck (SCCHN), the disease is locally advanced at presentation, and the 3- to 5-year survival rates remain below 30% (Veterans Affairs Laryngeal Cancer Study Group, 1991; Dimery and Hong, 1993; Vokes *et al*, 1993; Paccagnella *et al*, 1994; Lefebvre *et al*, 1996; Merlano *et al*, 1996). During the past 10 years, combined-modality approaches have been developed in an effort to enhance locoreginal disease control, reduce distant metastatic spread and improve survival in patients with locally advanced or inoperable SCCHN. Randomised trials have shown that chemoradiotherapy and induction chemotherapy are effective in preserving organ function in a subset of patients by reducing the need for surgery of the primary tumour site in patients with resectable disease, and improving survival in patients with unresectable disease (Veterans Affairs Laryngeal Cancer Study Group, 1991; Paccagnella *et al*, 1994; Lefebvre *et al*, 1996; Merlano *et al*, 1996; Calais *et al*, 1999). The standard induction regimen consists of cisplatin and continuous-infusion 5-fluorouracil (5-FU) (PF) and has been associated with a complete response (CR) rate of about 30% in randomised trials (Kish *et al*, 1985; Veterans Affairs Laryngeal Cancer Study Group, 1991; Lefebvre *et al*, 1996). The survival of patients with stage III and IV disease remains poor despite these improvements.

Docetaxel is a new agent that has demonstrated significant activity against SCCHN. A number of studies have explored the combination of docetaxel, cisplatin and 5-FU (TPF) in SCCHN, with promising results. The overall response rate with the three drugs in previously untreated, advanced SCCHN was approximately 64–93% (Janinis *et al*, 1997; Colevas *et al*, 1999; Schrijvers *et al*, 1999a). Different investigators also examined the feasibility of combination of docetaxel and radiotherapy. Koukourakis *et al* (1999) tested in a phase I setting the combination of docetaxel, irinotecan and conventional radiotherapy. With combination of weekly docetaxel 20 mg m^−2^ and irinotecan 25 mg m^−2^, the overall response rate in 12 patients with locally advanced head and neck cancer was 100%, with 75% CRs.

Here, we report the results of aggressive concurrent chemoradiotherapy with TPF in patients with locally advanced SCCHN. The main end points of this study were the toxicity of this concurrent chemoradiotherapy and preliminary assessment of the efficacy of this regimen in patients with advanced and untreated SCCHN.

## PATIENTS AND METHODS

### Patient population

Patients were selected if they had histologically or cytologically confirmed SCCHN, at least one unidimensionally measurable lesion, and stage III or IV disease without evidence of distant metastases. Patients with primary sites in the nasopharynx, mesopharynx, hypopharynx, larynx, oral cavity or paranasal sinus were eligible. Patients who had received previous chemotherapy, radiotherapy or surgery were excluded. Patients were ineligible if they had another cancer.

Patients were required to be from 20 to 75 years of age and have an Eastern Cooperative Oncology Group (ECOG) performance status (PS) of 0 or 1, life expectancy of at least 3 months, a WBC count of ⩾4000 cells *μ*l^−1^, an absolute neutrophil count (ANC) of ⩾2000 cells *μ*l^−1^, a platelet count of ⩾100 000 *μ*l^−1^, a haemoglobin level of ⩾9.5 g dl^−1^, AST, ALT and alkaline phosphatase levels below 2.5 times the upper limit of normal (ULN), total bilirubin and creatinine levels below than 1.5 times ULN, a BUN level below the ULN and a 24-h creatinine clearance rate of more than 60 ml min^−1^. Patients with significant cardiac arrhythmia or heart failure were ineligible. All patients provided written informed consent prior to enrollment in the study.

### Treatment schedule and dose escalation

Before this trial, we had carried out phase I trial of combined induction chemotherapy with docetaxel, cisplatin, and 5-FU for patients with locally advanced SCCHN (Tsukuda *et al*, 2003). The maximum-tolerated dose (MTD) of the regimen was docetaxel 70 mg m^−2^ (day 1), cisplatin 70 mg m^−2^ (day 4), and 5-FU 750 mg m^−2^ day^−1^ (days 1–5), and the regimen was safe and generally well tolerated and demonstrated good efficacy in patients with locally advanced SCCHN. Based on that previous study, we decided the initial dose level of this study. This study was concurrent chemoradiotherapy, and toxicities, such as mucositis, leukocytopenia and neutropenia, were thought to be more severe than induction chemotherapy, so that we reduced the initial dose level of docetaxel and 5-FU.

Table 1

**Table 1:** Dose levels in phase I trial of concurrent chemoradiotherapy docetaxel/cisplatin/5-fluorouracil in SCCHN

	**Dose (mg m^−2^ day^−1^)**		
**Dose level**	**Docetaxel (day 1)**	**Cisplatin (day 4)**	**5-Fluorouracil (days 1–5)**
2	70	70	600
1^a^	60	70	600
0	60	60	600
−1	50	60	600

a Starting dose level. Courses were repeated every 4 weeks.

shows the dose-escalation levels. The initial dose level (level 1) was docetaxel 60 mg m^−2^ (day 1), cisplatin 70 mg m^−2^ (day 4) and 5-FU 600 mg m^−2^ day^−1^ (days 1–5). The administration schedule is shown in Figure 1

**Figure 1:**
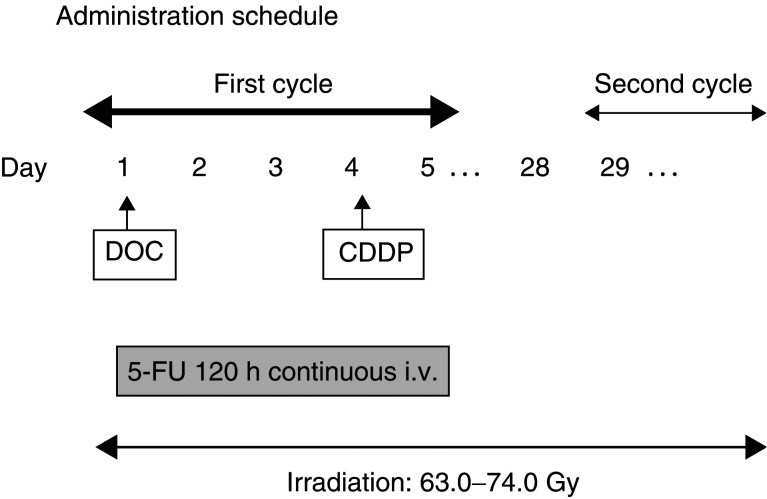
Administration schedule of docetaxel (DOC), cisplatin (CDDP), and 5-fluorouracil (5-FU) in phase I study of patients with advanced squamous cell carcinoma of the head and neck. i.v.=intravenous infusion.

. Docetaxel was administered intravenously over 1 h on day 1. More than 1 h after completion of the docetaxel infusion, 5-FU on days 1–5 was delivered by continuous intravenous infusion with 3.5 l of NS per day. Cisplatin was administered intravenously on day 4. Patients received ramosetron 0.3 mg and dexamethasone 8 mg intravenously on days 4–8 of chemotherapy. Two cycles of chemotherapy were repeated every 4 weeks.

Radiotherapy (1.8–2.0 Gy fraction^−1^ day^−1^), administered 5 days per week, was delivered to the primary tumour site and neck and was targeted to begin on the first day of chemotherapy, day 1. The mean total dose to the primary tumour site and neck lymph nodes was 67.8 Gy (range, 63.0–74.0 Gy). Every effort was made to continue the radiation on schedule. Subcutaneous G-CSF 100 *μ*g body^−1^ day^−1^ was injected if the neutrophil count was less than 1000 cells ul^−1^ after chemotherapy.

Retreatment on day 29 required ANC of ⩾2000 cells *μ*l^−1^, a platelet count of ⩾100 000 *μ*l^−1^, a haemoglobin level of ⩾9.5 g dl^−1^, AST, ALT and alkaline phosphatase levels below 2.5 times the ULN, a 24-h creatinine clearance rate of more than 50 ml min^−1^ and resolution of all other nonhaematological toxicities (except alopecia, musculoskeletal pain and fatigue) to be baseline or less than grade 1. If there were some toxicities as above, cycle 2 chemotherapy was delayed, and if the delay exceeded 14 days, the patient was removed from the study.

Patients were monitored for toxicity (medical interview, physical examination and complete blood cell counts) during treatment. Blood and urine chemistries were performed 2 or 3 times a week.

The MTD was defined as follows. If all three patients at any dose level experienced a dose-limiting toxicity (DLT), subsequent patients were treated at the next lower dose level. If two out of three experienced a DLT, the prior dose level was defined as the MTD. If one out of three patients experienced a DLT, three additional patients were added at that dose level. If more than three out of six experienced a DLT, then the prior dose level was defined as the MTD. If less than three out of six experienced a DLT, subsequent patients were treated at the next higher dose level. If none of three patients experienced a DLT, subsequent patients were treated at the next higher dose level.

### Toxicity assessment

Toxicity was assessed once per cycle according to the 1998 National Cancer Institute Common Toxicity Criteria, version 2.0. Resolution of effects such as myelosuppression, mucositis, fever (>38.0°C) and other disorders was required prior to initiating the second treatment cycle. A DLT was defined as grade 4 mucositis that interrupts treatment for over 2 weeks, grade 4 thrombocytopenia, grade 2 nephrotoxicity, or grade 3 or 4 nonhaematologic toxicity, excluding alopecia, nausea, vomiting, anorexia and fatigue. In addition, grade 4 neutropenia, which was predicted to occur in most patients, was not considered a DLT, because these toxicities were clinically able to be managed by G-CSF support.

### Clinical response and further treatment

The clinical response was assessed for each patient according to the combined findings of CT, MRI and ultrasonic examinations at 3 weeks after the end of the chemoradiation therapy. The definitions of CR, partial response (PR), no change (NC) and progressive disease (PD) were based on the standard definitions established by the WHO (1979). Clinical responses to this chemoradiation therapy were confirmed by biopsies of the primary site in all cases. In the case of N1–3 lymph node disease, fine needle aspiration cytology of the neck lymph nodes was performed. Responses at the primary site and the regional nodes were scored separately, and the overall response was based on the worst of the two responses. Surgery of the primary tumour site was recommended for operable patients with resectable disease, who failed to achieve CR after the end of the chemoradiation therapy. Surgery was carried out routinely 4–6 weeks after the end of the chemoradiation therapy.

## RESULTS

### Patients

A total of 19 patients were entered in this trial between July 2002 and February 2003. The characteristics of the population are listed in Table 2

**Table 2:** Baseline patient characteristics

	**Dose level**			
**Patient characteristic**	**1 (*n*=3)**	**0 (*n*=8)**	**−1 (*n*=8)**	**Total (*n*=19)**
Male : female ratio	2 : 1	7 : 1	7 : 1	16 : 3
*Age* (years)				
Average	66.7	55.8	61.6	60.0
Range	(56–73)	(50–66)	(50–75)	(50–75)
				
*Performance status, no. of patients*				
0	2	6	6	14
1	1	2	2	5
				
*Primary site, no. of patients*				
Nasopharynx	1	1	1	3
Oropharynx	0	4	2	6
Hypopharynx	1	1	2	4
Larynx	1	0	2	3
Oral cavity	0	2	0	2
Paranasal sinus	0	0	1	1
				
*Stage, no. of patients*				
III	1	3	4	8
IVA	2	4	4	10
IVB	0	1	0	1

. In all, 16 patients were males and three females, and the average age was 60.0 years (range, 50–75 years). The PS (ECOG) was 0 in 14 patients and 1 in five patients. The primary disease sites were the nasopharynx (*n*=3), oropharynx (*n*=6), hypopharynx (*n*=4), larynx (*n*=3), oral cavity (*n*=2) and paranasal sinus (*n*=1). Eight patients had stage III disease, and the remaining 11 patients were stage IV. Nine patients (47%) had N2 (*n*=8) or N3 (*n*=1) status before treatment, and seven patients had N0 status.

### Toxicity

Table 3

**Table 3:** No. of patients with grade 3–4 toxicities and dose-limiting toxicities (DLTs)

**Dose level**	**1 (*n*=3)**		**0 (*n*=8)**		**−1 (*n*=8)**		**Total (*n*=19)**	
**Grade**	**3**	**4**	**3**	**4**	**3**	**4**	**3**	**4**
Leukocytopenia	2	1	5	1	1	0	8	2
Neutropenia	1	1	4	1	1	0	6	2
Anaemia	1	0	2	0	0	0	3	0
Thrombocytopenia	1	0	0	0	0	0	1	0
Elevated AST, ALT levels	1	0	1	0	0	0	2	0
Elevated creatinine level	1^a^	0	1^a^	0	0	0	2^a^	0
Mucositis	1	2 (1^b^)	5	3	1	3	7	8 (1^b^)

a Grade 2 renal toxicity (DLT).

b Grade 4 mucositis that interrupts radiation therapy for over 2 weeks (DLT).

shows the grade 3–4 toxicities and DLTs that occurred at each dose level. At level 1, all three patients experienced a DLT: grade 4 mucositis that interrupted the radiation therapy for over 2 weeks in one patient, grade 3 anaemia in one, grade 3 elevation of the AST level in one and grade 2 elevation of creatinine in one. Next, patients were treated at level 0, and one of three patients experienced a DLT; thus, three additional patients were added at level 0. Three of six experienced a DLT: grade 3 anaemia in two patients, grade 3 ALT elevation in one and grade 2 elevation of creatinine in one. Thus, the doses used at level 0 were deemed the MTDs of the regimen (i.e., docetaxel 60 mg m^−2^, cisplatin 60 mg m^−2^ and 5-FU 600 mg m^−2^ day^−1^). Additional patients were entered at levels 0 and −1.

All 19 patients were assessable for toxicity. Mucositis was the most common adverse effect observed, with grade 3 (*n*=7) and grade 4 (*n*=8) mucositis observed in 79% (15 out of 19) of the patients, and grade 4 mucositis that interrupted the radiation therapy for over 2 weeks was observed in one patient at level 1. Grade 3 (*n*=8) and grade 4 (*n*=2) leukocytopenia were observed in 53% (10 out of 19) of the patients, and grade 3 (*n*=6) and grade 4 (*n*=2) neutropenia were observed in 42% (8 out of 19). Grade 3 anaemia was observed in three patients, grade 3 ALT and AST elevation in two, grade 3 thrombocytopenia in one and grade 2 creatinine elevation in one.

### Responses

At 3 weeks following completion of the chemoradiotherapy, all patients underwent biopsies of the primary tumour and/or fine needle aspiration cytology with ultrasound technique of neck lesions to determine the pathological response. As can be seen in Table 4

**Table 4:** Clinical response at each dose level, and by primary site and metastatic lymph nodes

**Dose level (doses of docetaxel/cisplatin/5-FU)**	**No. of evaluable patients**	**Overall response**	**Site**	**CR**	**PR**	**NC**	**PD**	**NE**
1	3	CR 3	P	3				
(60/70/600)			N	2				1
								
0	8	CR 7	P	7	1			
(60/60/600)		PR 1	N	4	1			3
								
−1		CR 6	P	7	1			
(50/60/600)	8	PR 2	N	3	2			3
								
Total	19	CR 16	P	17	2			
		PR 3	N	9	3			7

P=primary site; N=metastatic lymph node; NE=not evaluable because N0 at baseline.

, the overall clinical response rate was 100% (19 out of 19) and the pathological CR was 84% (16 out of 19). The primary site CR was 89% (17 out of 19) and metastatic lymph node CR was 75% (nine out of 12). After the chemoradiation therapy, there were three PR patients. A patient with oral cavity cancer (T4N2c) assigned to dose level 0 had PR at both the primary site and metastatic lymph node. A patient with hypopharynx cancer (T3N2c) in the level −1 had CR at the primary site and PR at the metastatic lymph node. These two patients underwent surgery. One patient with cancer of the hypopharynx (T4N2c) in level −1, with metastatic retropharyngeal lymph nodes, had PR at the primary site and metastatic lymph node, and he refused surgery and received oral 5-FU (TS-1; 100 mg day^−1^) (Inuyama *et al*, 2001).

With a median follow-up of 8 months and a range 3–11 months as of July 2003, all 19 patients were alive and 18 patients had no evidence of disease.

## DISCUSSION

Multiple studies have demonstrated chemotherapy and radiation therapy to be highly effective in increasing the survival of patients with unresectable disease. Concurrent chemoradiotherapy and induction chemotherapy have been established as an appropriate standard of care for many patients with locally advanced SCCHN.

The present study was designed to establish the MTD, safety and efficacy of concurrent chemoradiotherapy, including docetaxel, cisplatin and 5-FU in patients with locally advanced SCCHN. The MTD was identified as docetaxel 60 mg m^−2^, cisplatin 60 mg m^−2^ and 5-FU 600 mg m^−2^ day^−1^. The regimen was generally well tolerated and resulted in a high overall response rate of 100% and CR rate of 84%.

Many studies have explored the PF chemotherapy followed by radiation in SCCHN. The most comparable phase III trial of PF chemotherapy followed by radiation was the European Organization for Research and Treatment of Cancer (EORTC) Hypopharynx Trial (Lefebvre *et al*, 1996). In the EORTC trial, patients were treated with chemotherapy consisting of cisplatin 100 mg m^−2^ on day 1 and 5-FU 1000 mg m^−2^ day^−1^ on days 1–5 every 4 weeks for three cycles, and afterwards they were treated with irradiation (70 Gy). In this trial, 51% showed a clinical CR, and they showed that PF chemotherapy followed by radiation was effective in increasing the overall survival in unresectable disease, compared with radiotherapy alone. Using PF, Taylor *et al* (1994) compared concurrent chemoradiotherapy and chemotherapy followed by radiation for toxicity and efficacy in patients with SCCHN. This was a randomised trial between cisplatin 60 mg m^−2^ on day 1 plus 5-FU 800 mg m^−2^ on days 1–5 plus radiation 2 Gy on days 1–5, repeated every other week for seven cycles, *vs* cisplatin 100 mg m^−2^ on day 1 plus 5-FU 1.0 g m^−2^ on days 1–5, repeated every 3 weeks for three cycles, followed by 70 Gy of radiation for 7–8 weeks. After all treatments, CR did not differ between the two groups (52% in concurrent chemoradiotherapy and 50% in chemotherapy followed by radiation). However, in terms of the overall response rates, the concurrent chemoradiotherapy was better than the chemotherapy followed by radiation (*P*=0.003) (93% in concurrent chemoradiotherapy and 78% in chemotherapy followed by radiation), and fewer patients with no change or progression after concurrent treatment. Severe and worse toxic events were similar between the treatment programmes. The concurrent chemoradiotherapy with PF achieved improved disease control, predominantly of regional disease, compared with chemotherapy followed by radiation.

Docetaxel was shown to be an effective agent in SCCHN in multiple phase II studies (Forastiere *et al*, 1993, 1994; Catimel *et al*, 1994; Fujii *et al*, 1995; Couteau *et al*, 1996; Dreyfuss *et al*, 1996). Its mechanism of action and side effects are different from both cisplatin and 5-FU. Myelotoxicity is the DLT for the taxanes, whereas myelotoxicity of PF is mild.

Different investigators also examined the combination of docetaxel and radiotherapy. Mauer *et al* (1998) showed that it was possible to combine doses of docetaxel up to 20 mg m^−2^ week^−1^ with conventional radiotherapy of 1.8–2.0 Gy day^−1^ to a total dose of 60 Gy in patients irradiated at the thorax. Toxicity was mild, with oesophagitis and neutropenia as dose-limiting toxicities.

Some studies explored combination of docetaxel and PF for treatment of advanced SCCHN. Schrijvers *et al* (1999b) used cisplatin 75 or 100 mg m^−2^, docetaxel 75 mg m^−2^ and 5-day continuous infusion of 5-FU 750 mg m^−2^ day^−1^. The overall response rate was 80%, none of which were CRs. Janinis *et al* (2001) reported a 90% overall response rate and 20% CR rate with cisplatin 40 mg m^−2^ day^−1^ for 2 days, docetaxel 80 mg m^−2^ and 3-day continuous infusion of 5-FU 1000 mg m^−2^ day^−1^. Furthermore, Posner *et al* (2001), using cisplatin 75 or 100 mg m^−2^, docetaxel 75 mg m^−2^ and 4-day continuous infusion of 5-FU 1000 mg m^−2^ day^−1^, reported an overall response rate of 93% and CR rate of 40%.

A number of studies explored TPF chemotherapy followed by radiation for SCCHN. Colevas *et al* (1999) studied combination chemotherapy with docetaxel (60 mg m^−2^) on day 1, cisplatin (31.25 mg m^−2^ day^−1^), 5-FU (700 mg m^−2^ day^−1^) and leucovorin (500 mg m^−2^ day^−1^) on days 1–4 as a continuous infusion (TPFL-4), followed by radiation for SCCHN. Cycles were repeated every four weeks for three cycles. The overall RR was 93%, and the CR rate was 63%. The major toxicities were mucositis, nausea, neutropenia, anorexia; nephropathy, neuropathy and diarrhoea. This study of concurrent chemoradiotherapy with TPF achieved an overall RR of 100%. All patients underwent biopsy, and the pathological CR rate was 84%. The response rates and pathological CR rates of this concurrent chemoradiotherapy with TPF were; better than those seen with TPF chemotherapy followed by radiation, although comparisons between these studies are difficult because of the variable drug doses.

In the present study, mucositis was the most common adverse effect observed, with grade 3 (*n*=7) and grade 4 (*n*=8) mucositis observed in 79% of patients. Calais *et al* (1999) showed the incidence of grade 3 and 4 mucositis in the concurrent chemoradiotherapy with PF was 71% and higher than with radiotherapy only (39%). The incidence of mucositis was not so different between concurrent chemoradiotherapy with TPF and PF. Grade 3 (*n*=8) and grade 4 (*n*=2) leukocytopenia were observed in 53% of the patients, grade 3 (*n*=6) and grade 4 (*n*=2) neutropenia in 42%, grade 3 anaemia in three patients and grade 3 thrombocytopenia in one patient. There was a significant difference in haematologic toxicity between concurrent chemoradiotherapy with TPF and PF, and TPF therapy results in an early, brief and consistent neutropenia. Grade 4 mucositis that interrupted radiation therapy for over 2 weeks was observed in one patient at level 1, but all 19 patients were assessable for toxicity. These findings suggest that the toxicity of the concurrent chemoradiotherapy with TPF compares favourably with concurrent chemoradiotherapy with PF chemotherapy.

Before this trial, we had carried out phase I trial of combined induction chemotherapy with docetaxel, cisplatin and 5-FU for patients with locally advanced SCCHN (Tsukuda *et al*, 2003). The MTD of the regimen was docetaxel 70 mg m^−2^ (day 1), cisplatin 70 mg m^−2^ (day 4), and 5-FU 750 mg m^−2^ day^−1^ (days 1–5). Based on that previous study, we decided the initial dose level of this study. This study was concurrent chemoradiotherapy, and toxicities, such as mucositis, leukocytopenia, neutropenia, were thought to be more severe than induction chemotherapy; therefore, we reduced the initial dose level of docetaxel and 5-FU, and the initial dose level (level 1) was decided to be docetaxel 60 mg m^−2^ (day 1), cisplatin 70 mg m^−2^ (day 4) and 5-FU 600 mg m^−2^ day^−1^ (days 1–5). This study was constructed as a dose-escalation study but ended as a DE-escalation study. We underestimated the toxicities of radiation.

This concurrent chemoradiotherapy with TPF showed major antitumour activity with manageable toxicity as treatment of SCCHN patients. The high CR rate justifies further evaluation of this chemoradiotherapy combination. Based on the high response rate shown in this study, we are conducting a randomised comparison of concurrent chemotherapy with TPF *vs* concurrent chemoradiotherapy with CDDP, 5-FU, methotrexate and leucovorin.
